# Letter from Adjunct Professor Martins, *of the Faculty of Medicine of Paris*

**Published:** 1839-06-29

**Authors:** 


### FOREIGN CORRESPONDENCE.

### LETTER FROM ADJUNCT PROFESSOR MARTINS, *of the Faculty of Medicine of Paris.*

### No. II.

*On the method of Measuring the Temperature of the Human Body in Various Diseases.*

To the Editors of the Medical Examiner.

PARIS, April 27th, 1839.

Any inflamed tissue or organ communicates the sensation of heat. In a phlegmon or an erysipelas, it may be appreciated by the hand of the physician; in an internal inflammation, it is distressingly felt by the suffering patient. In some affections, there is the sensation of cold. In the first stage of an intermittent fever, the patient feels it; in an attack of cholera, the physician. It may naturally be supposed, that the observers of these phenomena have been anxious to measure them with exactness; but their attempts have heretofore failed, or have been unattended with accurate results. The causes of these failures have been:—1st, the imperfection of the instruments employed; 2d, a want of experience in the observers who have attempted to use them. For the last ten years, however, the subject in question has occupied the attention of a gentleman of skill and science, M. Walferdin. He has published only a few isolated results on the topic, but he has been so kind as to place at my disposal the following important views and facts which I am about to lay before your readers, and which have never before been made public. They are, in my opinion, entitled to the highest consideration of the physician, as well to spare him useless labour, as to point out to him the only path by which this important point of physiology and pathology can be advanced.

Ordinary thermometers serve to distinguish only striking differences of temperature, inasmuch as they are ordinarily divided into degrees, the extent of which does not exceed a line; and, in fact, the means of constructing the instruments otherwise are unknown, without giving them an inconvenient size. It is hence difficult to appreciate, with any thing like exactness, above a quarter of a degree. These defects are notorious, and acknowleged by all men of science. But, besides these, there are others which are less generally appreciated; for it is not generally understood, that the indices used to mark the degrees are themselves exceedingly inaccurate.

In the first place, the tube of a thermometer is never perfectly cylindrical. To satisfy yourselves of this fact, you have only to introduce a small quantity of mercury into a tube, and, upon examining it with a magnifying glass, you will not find it of the same length in any part of the tube. But, how do the makers of thermometers attempt to obviate this defect? In the following manner: They compare the thermometer, which they are graduating, with a standard thermometer, at zero, at 15°, at 30°, &c. These points are, therefore, exact; that is to say, when the mercury is at 15°, that temperature is accurately marked. Not so, however, between zero and 15°. Let us, for example, suppose that the cylinder of the tube is greater between 6° and 8° (centigrade,) than elsewhere; it follows, that the marks of these three degrees ought to be less distant from each other than the rest, since the same quantity of mercury will occupy a much smaller space: but this is not actually the case in the instrument, all the degrees of which are equal, and, consequently, inexact. It follows, therefore, that for a thermometer to be an accurate instrument, it should be graduated from degree to degree, which is quite out of the question.

We shall next proceed to point out another defect, of no less importance than the former, which necessarily belongs to all ordinary thermometers. When the thermometer to be constructed is compared with the standard thermometer, it scarcely ever happens that these two instruments have bulbs of equal capacity. What is the consequence of this difference of capacity? Let us suppose, for example, that the bulb of the standard thermometer is the smaller: the two instruments are plunged into water of about the temparature of 15°, and the height to which the mercury rises in the thermometer to be graduated is marked 15°, because that cipher is indicated on the other instrument. But the fact is not borne in mind, that the bulb of the latter being much smaller, is operated upon more readily by the heat of the water, and reaches its temperature more rapidly than the bulb of the other; consequently, when the standard thermometer marks 15°, the height to which the mercury will have reached in the stem of the other, will actually correspond only to 14°, or 13°.5, or even 13°. But, it will be said, there is a very simple means of remedying this source of error: it will be accomplished by leaving the two thermometers long enough in the liquid for both to attain an equilibrium of temperature. But, in avoiding this, you fall into another error. A liquid never remains for a length of time at a fixed temperature; it is constantly either rising or falling, and one of the thermometers will always indicate these variations before or after the other, according as its bulb is smaller or larger.

How then has M. Walferdin avoided these defects, in the thermometers which he has contrived to measure animal temperature? In the first place, in order not to give his instruments too great a length, he constructs them so that the mercury does not escape from the bulb under 30°, (centigrade,) and that at 45° it reaches the extremity of the stem. The thermometer has, therefore, a range of 15°; its total length is from 6 to 7 inches, and is divided into 250 or 260 parts. Hence a degree of centigrade corresponds to about 16 or 17 divisions; and a half a division, (which is easily estimated,) gives you the 1-32d of a degree of centigrade. The bulb of these thermometers has the shape of an olive, and the walls of the tube are very thick, and of the same diameter as the bulb, although the mercurial column is quite capillary. It is hence easy to introduce the thermometer into the mouth, the anus, the urethra, of men and animals, without danger of its being broken. It has been seen that thermometers, of the kind described, indicate variations in the animal temperature of 1-32d of a degree, and I have already made use of them with success, to ascertain the influence of diet and of the seasons upon the animal temperature. I am waiting to publish these results, until I collect an amount of accurate observations.

To obviate the first inconvenience spoken of, the inequality in the diameters of the tubes of different thermometers, M. Walferdin compares his thermometer with a standard as perfect as possible, at points of, for example, five degrees of distance, from 30° to 45°, by plunging them into a large mass of liquid, the temperature of which is made to remain fixed for about ten minutes, by a method to be presently pointed out. He marks the number of divisions, which corresponds, for instance, to the space between 30° and 35°; then that which corresponds to the space between 35° and 40°, and so on. It is evident that, by this means, we altogether avoid the inconvenience arising from the inequality of the diameter of the tube in its whole length. In fact, there will be sometimes 16 divisions corresponding to a degree of centigrade, sometimes 16.5, or even 17, and sometimes but 15, and, according to the point observed, will be determined the value of a degree at the point.

M. Walferdin determines the fixedness of the temperature of the liquid employed to compare the instruments, by means of a little ingeniously-contrived thermometer, which he calls the *metastatic spirit-thermometer*. This is a little thermometer nearly filled with colourless alcohol, but in the bulb of which there is a globule of mercury. To ascertain that the temperature of any medium undergoes no variation, this little thermometer is plunged into it; it takes the temperature of the medium; is withdrawn; evaporation produces a slight fall of the temperature; it is then suddenly turned upside down; the globule of mercury becomes engaged in the capillary tube of the thermometer, which is immediately replunged into the medium. It now remains in the stem, but its undulations indicate the slightest variations in the temperature of the liquid. Mr. Walferdin makes use of a thermometer of this kind, the whole length of which is but a degree of centigrade, and, being divided into 500 parts, will point out variations of the $\frac{1}{500}$ of a degree. When this little thermometer remains without variation for five or ten minutes, it is certain that the temperature of the liquid has not changed, and, consequently, that the two thermometers are at the same degree of heat. Then, upon that which is to be graduated, is marked the height which the mercurial column has reached, and the degree is of course exactly denoted.

These purely scientific details are, perhaps, a little dry; but they will not, I trust, be without interest to readers who must be desirous of knowing how far they can rely upon the instruments they employ, and who will, therefore, pardon this digression into the domain of medical physics. My next letter shall be devoted to a more strictly medical subject.

